# Epidemiology of CKD Regression in Patients under Nephrology Care

**DOI:** 10.1371/journal.pone.0140138

**Published:** 2015-10-13

**Authors:** Silvio Borrelli, Daniela Leonardis, Roberto Minutolo, Paolo Chiodini, Luca De Nicola, Ciro Esposito, Francesca Mallamaci, Carmine Zoccali, Giuseppe Conte

**Affiliations:** 1 Nephrology Division at Second University of Naples, Naples, Italy; 2 Nephrology Diision.-Center of National Research-Institute of Biomedicine and Molecular Immunology Hospital, Reggio Calabria, Italy; 3 Division of Medical Statistics at Second University of Naples, Naples, Italy; 4 Division of Nephrology at IRCCS Maugeri Institute of Pavia, Pavia, Italy; Kaohsiung Medical University HospitalKaohsiung Medical University HospitalKaohsiung Medical University Hospital, TAIWAN

## Abstract

Chronic Kidney Disease (CKD) regression is considered as an infrequent renal outcome, limited to early stages, and associated with higher mortality. However, prevalence, prognosis and the clinical correlates of CKD regression remain undefined in the setting of nephrology care. This is a multicenter prospective study in 1418 patients with established CKD (eGFR: 60–15 ml/min/1.73m²) under nephrology care in 47 outpatient clinics in Italy from a least one year. We defined CKD regressors as a ΔGFR ≥0 ml/min/1.73 m^2^/year. ΔGFR was estimated as the absolute difference between eGFR measured at baseline and at follow up visit after 18–24 months, respectively. Outcomes were End Stage Renal Disease (ESRD) and overall-causes Mortality.391 patients (27.6%) were identified as regressors as they showed an eGFR increase between the baseline visit in the renal clinic and the follow up visit. In multivariate regression analyses the regressor status was not associated with CKD stage. Low proteinuria was the main factor associated with CKD regression, accounting *per se* for 48% of the likelihood of this outcome. Lower systolic blood pressure, higher BMI and absence of autosomal polycystic disease (PKD) were additional predictors of CKD regression. In regressors, ESRD risk was 72% lower (HR: 0.28; 95% CI 0.14–0.57; p<0.0001) while mortality risk did not differ from that in non-regressors (HR: 1.16; 95% CI 0.73–1.83; p = 0.540). Spline models showed that the reduction of ESRD risk associated with positive ΔGFR was attenuated in advanced CKD stage. CKD regression occurs in about one-fourth patients receiving renal care in nephrology units and correlates with low proteinuria, BP and the absence of PKD. This condition portends better renal prognosis, mostly in earlier CKD stages, with no excess risk for mortality.

## Introduction

Chronic kidney disease (CKD) is traditionally considered an unremittingly progressive disease and early identification of risk factors predicting faster CKD progression is the centerpiece of current guidelines [1]. CKD regression is considered as an uncommon outcome in these patients. However, solid evidence in experimental models and patients exists that renal damage may regress [2,3].

In the Afro-American Study of Kidney Disease (AASK) trial, a tiny minority (3.3%) of patients exhibited positive slopes of Iothalamate-measured GFR (+1.06 ml/min/1.73m^2^) which could not be explained by random measurement variation [4]; similarly, in a subgroup of 406 patients selected among the 1,269 enrolled in the NEPHROTEST study with at least three available ^51^Cr-EDTA renal clearance measurements, 15% of patients manifested a GFR improvement over time [5]. However, the peculiar risk profile of African Americans and the highly selected nature of the NEPHROTEST sub-cohort, in which 70% of participants with less than three GFR measurements were excluded, may limit the generalizability of these estimates of CKD regression. Similarly, these two studies provided only few data on the demographic, clinical and biochemical factors which associate with eGFR improvement over time [4,5]. More important, the prognostic role of CKD regression was not addressed in these studies. The issue is of major relevance because recent studies, primarily designed to assess the relationship between the decline of renal function and mortality, reported that CKD patients showing eGFR increase over follow up do exhibit a clear-cut trend toward higher mortality [6–9].

We therefore evaluated GFR changes over time in 1,418 adult patients with CKD stage 3–4 receiving renal care in the clinics networks of two cohort studies in Italy, the TArget Blood pressure LEvels (TABLE) study and Multiple intervention and AUdit in Renal diseases to Optimize care (MAURO) study [10,11]. The study is aimed at defining the prevalence of CKD regressors, correlates associated with this condition and the prognostic role of CKD regression for the risk of ESRD and mortality.

## Methods

### Study design and selection criteria

This is a multicenter cohort study involving forty-seven Italian renal clinics which participated into two cohorts studies [10,11]. Study protocol was in conformity with ethical guidelines of our institutions, and it was approved by ethics committees of Second University of Naples for TABLE study and of Center of National Research-Institute of Biomedicine and Molecular Immunology Hospital of Reggio Calabria for MAURO study; then was approved by local ethics committees of all nephrology units that participated to the study. Written informed consent was obtained from each patient. Participating centers shared renal care protocols adhering to International and National CKD Guidelines [12,13].

We pooled individual patients’ data of the two cohorts [14]. The first cohort was the TABLE cohort constituted by consecutive CKD patients with eGFR<60 ml/min/1.73m² enrolled in 25 renal clinics during 2003. All patients in this cohort had a fully recorded first visit at the same renal clinics with a diagnosis of CKD dating back at least 1 year before recruitment [10]. Main inclusion criteria were absence of acute changes of eGFR ≥30% in the six months before selection, kidney transplant, pregnancy and cancer or diseases in the terminal phase. The second cohort (MAURO) was composed of consecutive adult patients with serum creatinine 132–354 mmol/dl (if males) and 115–309 mmol/dl (if females), recruited between October 2005 and November 2007 in 22 renal clinics on the basis of selection criteria similar to TABLE study. As previously described, the main inclusion criteria of the MAURO study were: at least 2 measurements of of eGFR during the run-in period; non-acute or rapidly evolving renal disease; non transplanted, not pregnant, not affected by cancer or disease in a terminal phase [11].

Two phases were planned. In the first phase, from baseline to follow-up visit lasting 18–24 months, we ascertained the status of "regressor" by calculating the rate of change of kidney function over time (ΔGFR). This was estimated as the absolute difference of eGFR between the baseline and the follow-up visit (occurring in the period between 18 and 24 months from baseline visit) and expressed as ml/min/1.73 m^2^/year. Patients deceased or dropped out in the same period were excluded from analyses. In the second phase of the study, lasting from the follow-up visit to the date of death, ESRD or last available visit, we assessed the survival difference between regressors and non-regressors.

### Patients' classification

According to the CKD Prognosis Consortium [15], we selected in each cohort patients with eGFR 60–15 ml/min/1.73m^2^, with at least two measurements of eGFR and the last measurement obtained 18–24 months after baseline. We defined as “regressors” those patients who experienced a ΔGFR ≥0 ml/min/1.73 m^2^/year. Indeed, since a physiological decline of about -1 ml/min/1.73 m^2^/year has been demonstrated in healthy adults [16–18], stability of eGFR over time can be still considered as an improvement of kidney function. Those with ΔGFR <0 ml/min/1.73 m^2^/year or those who developed ESRD over the same follow up were classified as “non regressors”.

### Data Collection

At the baseline visit, we collected demographics, medical history, office BP, laboratory parameters, including 24-h-urinary proteinuria, and therapy. Cigarette smoking was dichotomized as current smoker and non-smoker. Office BP was measured according to recommendations by the European Society of Hypertension by using standard mercury sphygmomanometers [19].

Diabetes was defined by either self-reported history of diagnosis, use of hypoglycemic drugs, or a fasting glucose level>126 mg/dl. Cardiovascular disease (CVD) was defined as electrocardiography-documented angina, history of myocardial infarction, stroke, transient ischemic attack, intermittent claudication, and prior revascularization procedures. Laboratory parameters were measured by standard methods in the clinical laboratories of participating centers. Office BP, laboratory data and therapy were also collected at follow up visit. All laboratories adhered to regional or national quality control programs.

Estimated GFR was calculated using the Chronic Kidney Disease Epidemiology Collaboration (CKD-EPI) 2009 creatinine equation [20,21]; the creatinine measurement was not standardized to isotope dilution mass spectrometry, so that the creatinine concentrations were reduced by 5% (the established calibration factor) [22]. Twenty-four hour urine collection was repeated if the measured creatinine excretion rate was outside the 60% to 140% range of the value calculated according to Dwyer and Kenler [23].

### Outcome of survival analysis

Study endpoints were ESRD and all-cause mortality before ESRD. Follow-up for either outcome started after the follow-up visit and lasted until June 30, 2011. ESRD date was the beginning of chronic dialysis or pre-emptive kidney transplantation. All participating centers were requested to follow the same procedure: to ascertain ESRD/death of patient phone calls to family members were made by local investigator when the patient missed two or more planned visits. Information on death, obtained by the family, was verified by death certificates provided by primary care physician of the patient. ESRD was also verified by means of medical records or regional dialysis registry.

### Statistical analysis

Continuous variables were reported as either mean±SD or median and interquartile range (IQR) according to their distribution and tested by means of unpaired Student t-test or Mann-Whitney. Categorical variables were reported as percentages and compared by using Pearson chi-squared test.

Logistic regression was used to identify basal factors associated with the regressor status. The model was built a priori by including age, gender, diabetes, BMI, CVD, smoking, cause of CKD, systolic BP, CKD stage, serum uric acid, hemoglobin, phosphate, and 24h proteinuria. The contribution of each covariate to the model fit was estimated as percentage reduction of R^2^ value of the model resulting, from omitting each variable in turn from the full model. We calculated R^2^ values according to Tjur approach [24]. Nonlinear association between proteinuria and eGFR improvement was found, and, therefore, a restricted cubic spline was used by Harrell approach [25] with four knots placed a priori at clinically relevant values (0, 0.5, 1 and 3 g/24h of proteinuria).

Median follow-up was estimated by the inverse Kaplan-Meier approach [26]. We used the Cox models to estimate event-specific hazard ratios (HRs) and 95% confidence intervals (CIs) of the regressor status. HRs were adjusted by age, gender, diabetes, BMI, previous CVD, smoking, and systolic BP, uric acid, Hb, phosphate, cholesterol and 24h proteinuria measured at follow up visit. In order to attenuate the bias due to the regression to the mean [27], the model was adjusted for CKD stages at follow up visit (stage 1–3a as reference, stage 3b and stage 4–5) rather than for eGFR. The nonlinear relationship of ΔGFR and outcomes (either ESRD or death) was evaluated using restricted cubic splines separately in CKD stages at follow up visit. Specifically, we placed 5 knots at 0.050, 0.275, 0.500, 0.725, and 0.950 quantiles of the ΔGFR, according Harrell method [25].

We also calculated the kappa-statistic measure of agreement in patients classification (regressors/non-regressors) when using ΔGFR over time (two points) or ordinary least-squares regression slope (from 3 to 5 points); agreement was classified according to Landis and Koch [28]. The data were analyzed using SAS version 9.2 (SAS Inc., Cary, NC).

## Results

**Fig 1:**
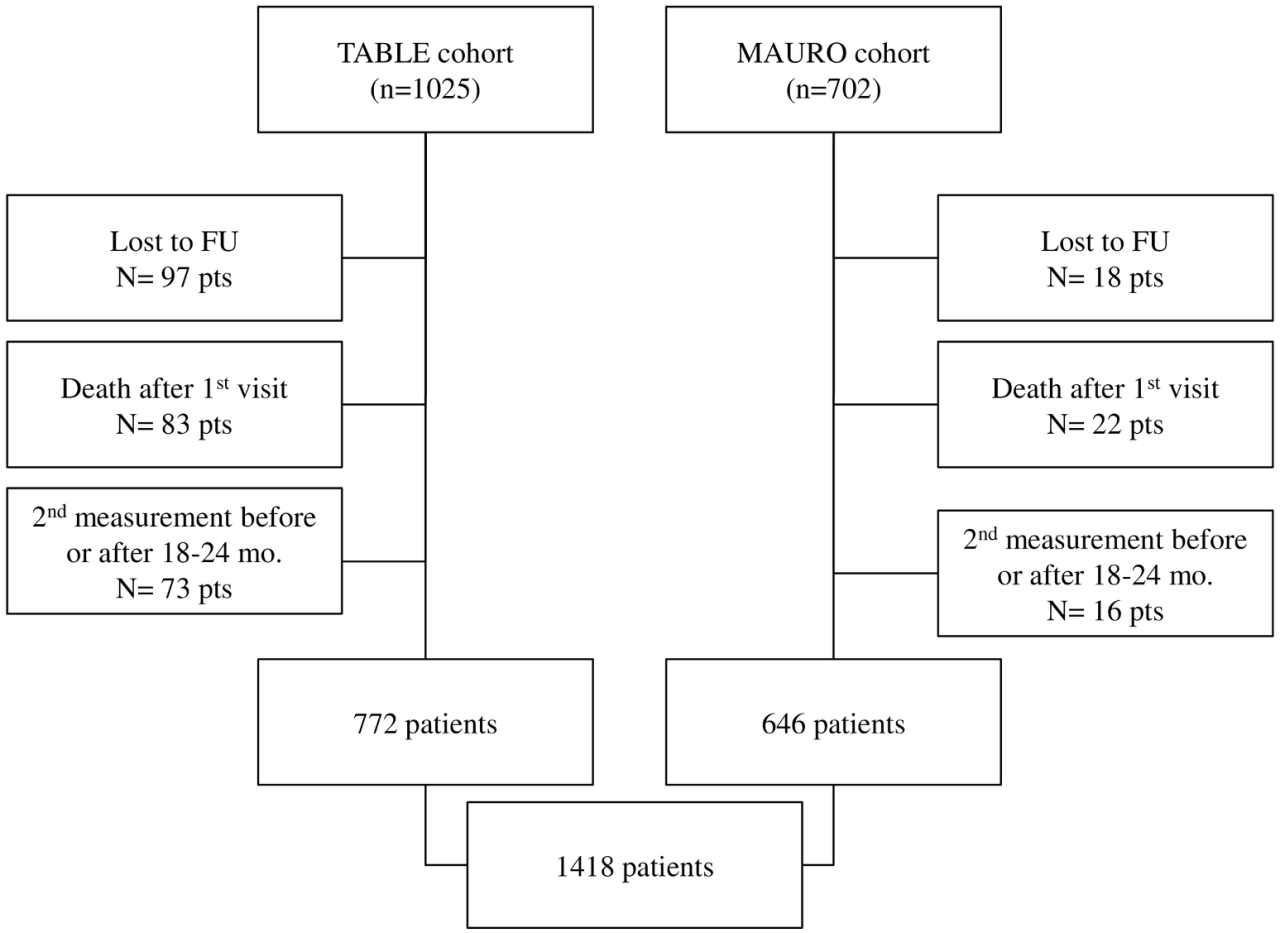
Selection algorithm of patients.

As described in Fig 1, out of the 1727 eligible patients, 1418 were studied. In particular, the first cohort of patients was extracted from a population of 1025 CKD patients from TABLE study. Among these, 83 were excluded because they died after the enrollment but before 2^nd^ eGFR assessment, 97 were excluded because they dropped out of the study and 73 were excluded because they had missing eGFR values in the time windows 18–24 months. The source population of the second cohort included 646 CKD patients (Fig 1). We excluded 18 because they dropped out of the study, 22 because they died after the enrollment but before 2^nd^ eGFR assessment, and 16 because they had the second eGFR measurement before or after 18–24 months. Therefore, 1418 CKD patients were available for the data analysis. The comparison of baseline characteristics of 105 patients that died during this phase showed an older age (75.0±9.5 years) and greater prevalence of cardiovascular disease (48.6%) than that recorded in patients included into the study.

All enrolled patients were Caucasian. They were characterized by a high proportion of older age (53.8% over 65 y), diabetes, previous CVD and smoking. At baseline, distribution of patients in CKD stage 3a, 3b, and 4 was 23.5%, 36.5% and 40.0%, respectively. The vast majority of patients (N = 1347, 94%) were on antihypertensive treatment: 23% were treated with one drug, 34% with two drugs, and 43% with three or more drugs. 1262 patients were treated with angiotensin-converting enzyme inhibitors and/or angiotenstin II blockers. Statins were used in one quarter of cohort, whereas use of fibrates was trivial (N = 8).

During the interval between baseline and follow-up visit (median: 23 months IQR 22–24), median ΔGFR was -1.7 ml/min/1.73m^2^/year (IQR from -4.6 to +0.8) for the whole cohort (Table 1), without significant differences among the three stages (CKD-3a:-1.4 ml/min/1.73m^2^/year, IQR from -5.7 to 2.2; CKD-3b: -1.5 ml/min/1.73m^2^/year, IQR from -4.5 to 0.9; CKD-4: -2.0 ml/min/1.73 m^2^/year, IQR from -4.1 to -0.0, P = 0.246). On the basis of ΔGFR, we classified 391 patients as regressors (27.6%, 95%CI 25.3–30.0) and 1,027 as non-regressors (73.4%, 95%CI 70.1–74.8), including 111 patients who progressed to ESRD between baseline and follow up visit. In a sensitivity analysis, we re-assessed the prevalence of regressors in a nested cohort of 1154 patients (82% of the whole cohort) with at least three eGFR measurements in 24 months (range: 3–5) by using the ordinary least-squares regression slope. In this nested cohort, the prevalence of regressors was 29.6% with almost perfect agreement with the simultaneous two-points estimate in the whole cohort (Cohen’s Kappa: 0.88; P<0.0001).

**Table 1:** Main demographic, clinical and therapeutic features of patients stratified by regressor status.

	Overall	Regressors	Non-regressors	P
	(N = 1418)	(N = 391)	(N = 1027)	
ΔGFR (*ml/min/yr*)	-1.7 (-4.6/0.8)*	2.4 (1.1/4.8)	-3.2(-5.4/-1.4)**	
Age (*years*)	63.9±12.2	63.7±11.7	63.9±12.3	0.773
Male Gender (%)	59.0	61.5	58.1	0.227
Diabetes (%)	29.2	24.9	30.8	0.032
Smokers (*%*)	24.2	26.3	23.4	0.222
History of CVD (*%*)	29.9	27.5	30.0	0.374
BMI (*kg/m* ^*2*^)	27.8±4.7	28.1±4.7	27.7±4.7	0.096
Renal Disease (*%*)				0.001
PKD	6.1	2.1	7.7	
Diabetic nephropathy	10.6	8.2	11.6	
Hypertensive nephropathy	19.4	20.6	18.9	
Glomerulonephritis	11.0	11.1	11.0	
Other	17.3	18.9	16.7	
Unknown	35.6	39.0	34.0	
Systolic BP (*mmHg*)	137±18	133±17	138±18	<0.0001
Diastolic BP (*mmHg*)	79±11	78±11	80±11	0.005
eGFR (*ml/min/1*.*73m* ^*2*^)	35±12	38±12	34±12	<0.0001
Proteinuria (*g/24 h*)	0.5 (0.2–1.1)	0.3(0.1–0.7)	0.6 (0.2–1.4)	<0.0001
Serum Albumin (*g/dl*)	4.0±0.5	4.1±0.5	4.0±0.5	0.003
Phosphate (*mg/dl*)	3.7±0.7	3.7±0.8	3.8±0.7	0.017
Uric Acid (*mg/dl*)	6.4±1.7	6.5±1.8	6.3±1.7	0.130
Hemoglobin (*g/dl*)	12.9±1.8	13.1±1.8	12.8±1.8	0.001
Cholesterol (*mg/dl*)	194±42	193±40	194±43	0.793
Antihypertensives (n)	2.2±1.1	2.2±1.1	2.2±1.1	0.381
ACEIs/ARBs (*%*)	81.8	81.3	81.9	0.797
ESA (*%*)	8.8	6.9	9.3	0.140
Statin (%)	24.8	27.9	23.8	0.245

Data are mean±SD, percent or median (IQR).

*delta eGFR was calculated in 1307 patients due to the exclusion of 111 patients who developed ESRD between baseline and follow up visit.

** delta eGFR was calculated in 916 patients due to the exclusion of 111 patients who developed ESRD between baseline and follow up visit.

Abbreviations: BMI, body mass index; CVD, cardiovascular disease; eGFR, glomerular filtration rate estimated by the 4-variable MDRD equation; PKD, polycystic kidney disease; BP, blood pressure. ACEi, Angiotensin converting enzyme inhibitor; ARB, Angiotensin II receptor blocker; ESA, erythropoiesis stimulating agents.

Regressors were characterized by a lower prevalence of diabetic nephropathy and PKD, lower BP, proteinuria and serum phosphate and higher serum albumin and hemoglobin (Table 1). The prevalence of regressors from CKD stage 3a, 3b and 4 was 36.0%, 29.2% and 21.1%, respectively (P<0.0001).

At the follow up visit, among non-regressors, serum phosphate increased while BP, hemoglobin, cholesterol and proteinuria declined. Of note, in regressors we observed a slight but significant increment of proteinuria (Table 2). Median values of delta proteinuria were 0 g/day (IQR form -0.13 to 0.28) and 0 g/day (IQR from -0.44 to 0.26), respectively for regressors and non-regressors (p = 0.009).

**Table 2:** Differences of clinical and laboratory parameters between baseline and follow up visit in regressors and non-regressors.

	*Regressors*			*Non-regressors*		
	Baseline	FU visit	P	Baseline	FU visit	P
Systolic BP (*mmHg*)	134±17	133±15	0.399	137±18^#^	134±17	<0.0001
Diastolic BP (*mmHg*)	78±11	77±9	0.187	80±11^§^	78±10	0.0002
eGFR(*ml/min/1*.*73m* ^*2*^)	38±12	45±16	-	35±12^#^	28±12*	-
Proteinuria(*g/24 h*)	0.3(0.1–0.7)	0.4(0.1–0.8)	0.023	0.6 (0.2–1.4)*	0.5(0.2–1.1)*	0.004
Serum Albumin (*g/dl*)	4.1±0.5	4.1±0.5	0.802	4.0±0.5^#^	4.0±0.5^§^	0.936
Phosphate(*mg/dl*)	3.7±0.8	3.7±0.9	0.197	3.7±0.7	3.9±0.9^§^	<0.0001
Uric Acid (*mg/dl*)	6.5±1.8	6.5±1.8	0.817	6.3±1.7	6.4±1.7	0.222
Hemoglobin(*g/dl*)	13.1±1.8	13.0±1.8	0.488	12.9±1.7	12.6±1.7*	<0.0001
Cholesterol (*mg/dl*)	193±40	184±39	0.002	194±42	185±44	<0.0001

*P<0.0001 vs. regressors;

^#^ p<0.001 vs. regressors;

^§^p<0.05 vs. regressors.

Abbreviations: FU: Follow up; eGFR, glomerular filtration rate estimated by the 4-variable MDRD equation; BP, blood pressure.

**Fig 2:**
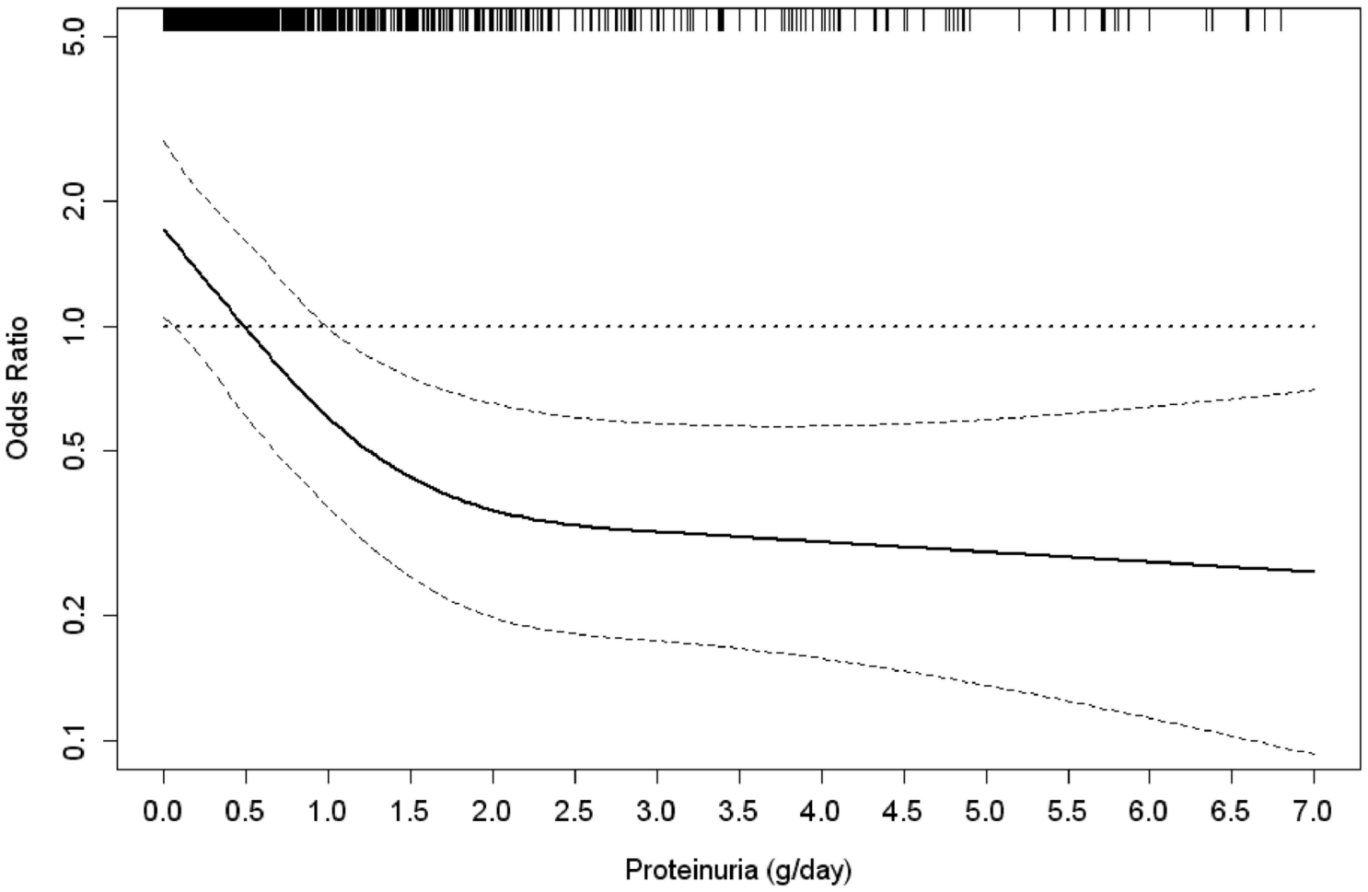
Plots of odds ratios and 95% confidence intervals (as indicated by the curvilinear dash lines) for likelihood of being regressor by level of proteinuria as continuous variable (reference level, 0.5g/24h). The distribution of observations is shown as a rug plot on the x axis.

Predictors of regressor status are reported in Table 3. Patients with higher BMI and lower levels of proteinuria and systolic BP were more likely regressors while patients with diagnosis of PKD had a lower probability of being regressors. In this analysis, more advanced CKD (stage 3b and stage 4) was not significantly associated to CKD regression (Table 3). When estimating the hierarchy of prognostic factors by R² reduction, low proteinuria emerged as the strongest factor associated with eGFR improvement, accounting *per se* for 48% of the variance of the model. The non-linear relationship between proteinuria and the odds of the regressor status is depicted in Fig 2. The figure shows an exponential decrease of the likelihood of being regressor from lowest levels of proteinuria up to about 2 g/day with a plateau for values >2 g/day. Furthermore, independent of proteinuria, PKD was associated with the lowest odds of CKD regression and this accounted for 20% of model variance.

**Table 3:** Logistic regression analysis estimating factors associated with the probability of being regressor and estimated contribution of each determinant to model fit (% R^2^ reduction).

	OR (95%)	P	% R^2^ reduction
Age (5 *years*)	0.98 (0.93–1.05)	0.622	0
Male Gender (*yes versus not*)	1.27 (0.94–1.71)	0.127	2
Diabetes (*yes versus not*)	0.83 (0.58–1.20)	0.328	1
Smokers (*yes versus not*)	1.28 (0.94–1.74)	0.116	2
CVD (*yes versus not*)	0.95 (0.71–1.28)	0.742	0
BMI (1 kg/m^2^)	1.05 (1.02–1.08)	0.001	8
Renal Disease			20
Unknown	Ref.		
Diabetic nephropathy	0.95 (0.55–1.65)	0.863	
Hypertensive nephropathy	0.96 (0.68–1.38)	0.841	
Glomerulonephritis	1.06 (0.67–1.70)	0.795	
Other	0.96 (0.66–1.38)	0.806	
ADPKD	0.17 (0.08–0.40)	<0.0001	
CKD Stages			2
3a	Ref.		
3b	0.90 (0.64–1.27)	0.553	
4	0.73 (0.53–1.09)	0.106	
Systolic BP (5 mmHg)	0.94 (0.901–0.98)	0.005	7
Phosphate (mg/dl)	1.05 (0.87–1.27)	0.635	0
Uric Acid (mg/dl)	1.05 (0.98–1.14)	0.186	0
Hemoglobin (g/dl)	1.03 (0.95–1.12)	0.502	0
ACEi and/or ARB use (*yes versus not*)	0.89 (0.64–1.24)	0.490	0
Proteinuria (mg/day)	—*	<0.0001	48

*Non linear effect (p<0.0001) as shown in Fig 2.

Abbreviations: BMI, body mass index; CV, cardiovascular; ADPKD, autosomal dominant polycystic kidney disease; CKD, Chronic Kidney Disease; BP, blood pressure. ACEi, Angiotensin converting enzyme inhibitor; ARB, Angiotensin II receptor blocker; ESA, erythropoiesis stimulating agents.

### Survival analysis

After the follow up visit (i.e., 18–24 months after baseline), 1307 patients were followed up for median time of 2.1 years (IQR: 1.0–3.2) to investigate the relationship of regressor status with the risk of ESRD and death. During this period, 173 ESRD events (all initiation of chronic dialysis treatment) and 110 deaths before ESRD occurred. Incidence rates were 7.6/100 patient-year (95% CI, 6.6 to 8.7) for ESRD and 3.6/100 patient-year (95% CI, 3.0 to 4.4) for death before ESRD.

Incidence rate for ESRD was markedly lower in regressors than in non-regressors (1.1 per 100 patient-year, 95% CI 0.6–2.2 and 10.4 per 100 patient-year, 95% CI 9.0–12.0, respectively) while no difference was detected for incidence of death (3.7 per 100 patient-year 95% CI, 2.5–5.2 and 3.7 per 100 patient-year, 95% CI 3.0–4.6 in regressors and non-regressors, respectively, P = 0.906). As compared to non regressors, ESRD risk was 72% lower in the subgroup of regressors while mortality risk did not differ (Table 4). A significant effect of CKD stage was detected for the risk of ESRD but not for mortality (Table 4). No interaction was found between regressors and CKD stage for both ESRD and all-cause death.

**Table 4:** Cox analysis estimating the adjusted risk for ESRD and all-causes death of patients on the basis of regressor status and eGFR levels at the follow up visit.

	HR	95%CI	P
**ESRD**			
Regressors	0.28	0.14–0.57	<0.0001
CKD stages			
eGFR >45 ml/min/1.73m^2^	1.00		
eGFR 30–45 ml/min/1.73m^2^	2.10	0.58–7.54	0.255
eGFR <30 ml/min/1.73m^2^	15.58	4.84–50.18	<0.0001
**All-cause death**			
Regressors	1.16	0.73–1.83	0.540
CKD stages			
eGFR >45 ml/min/1.73m^2^	1.00		
eGFR 30–45 ml/min/1.73m^2^	0.87	0.44–1.69	0.674
eGFR <30 ml/min/1.73m^2^	1.69	0.91–3.14	0.095

Analysis is adjusted for age, gender, diabetes, cardiovascular diseases, BMI, Systolic Blood Pressure, serum levels of phosphate, hemoglobin, uric acid, cholesterol and 24h proteinuria measured at follow up visit.

**Fig 3:**
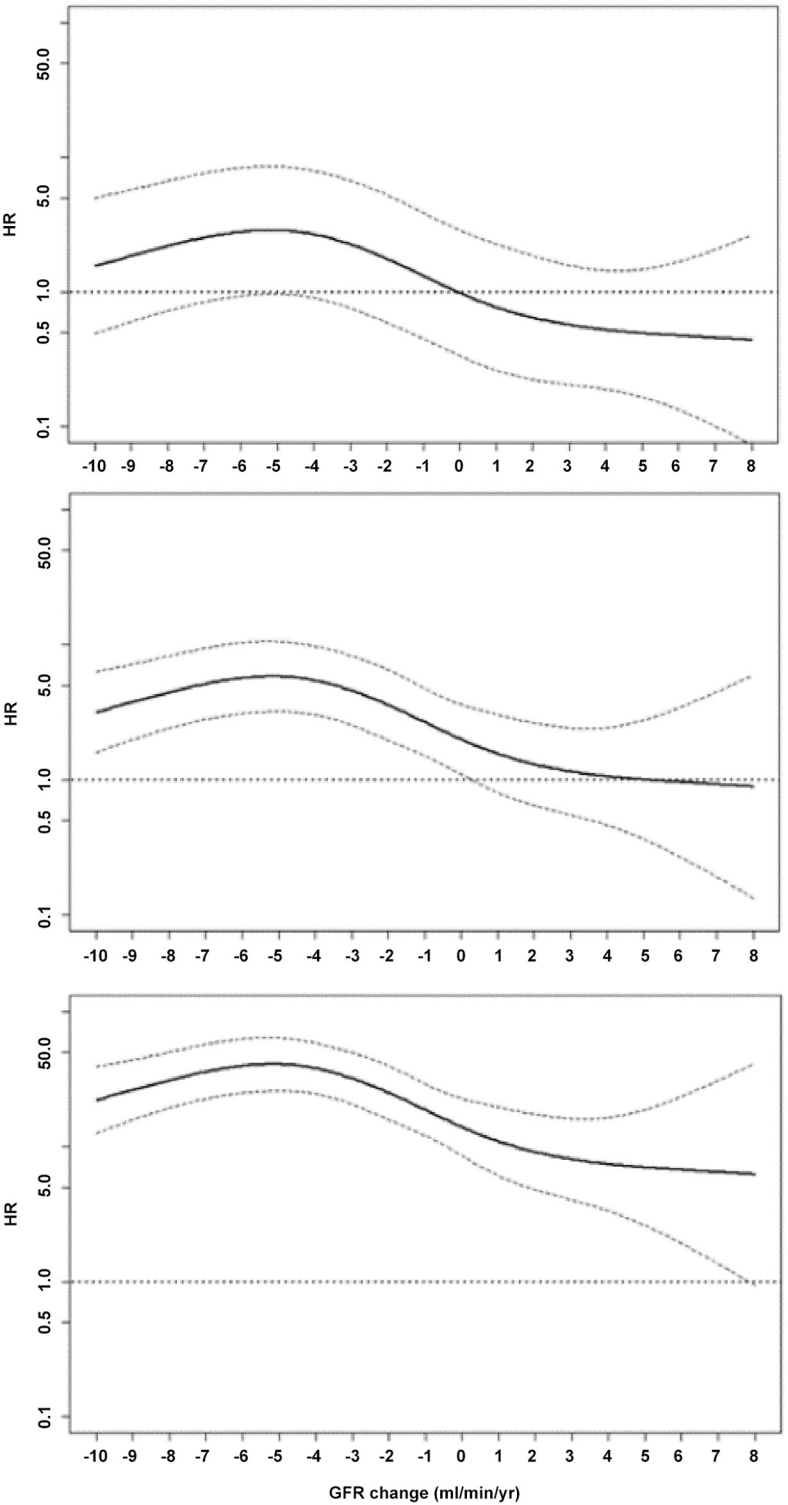
Restricted cubic splines to evaluate the non-linear relationship of eGFR change with ESRD at each GFR level: above 45 ml/min/1.73 m^2^ (top), between 30 and 45 ml/min/1.73 m^2^ (middle) and below 30 ml/min/1.73 m^2^ (bottom). The reference for the three GFR strata is 0 ml/min/1.73m^2^ when ‎GFR level >45 ml/min/1.73 m^2^ (top). Spline model is adjusted for age, gender, diabetes, BMI, previous CV disease, smoking, systolic BP, uric acid, hemoglobin, phosphate, cholesterol and 24h proteinuria measured at the follow up visit.

When we included in the Cox models ΔGFR as continuous variable the relationship with ESRD was not linear; therefore, we performed additional analyses using restricted cubic spline for each CKD stage at follow up visit. The association between ΔGFR and renal risk was modified by stage of disease. As depicted in Fig 3, in fact, improvement of renal function (positive ΔGFR) was associated to a greater risk of ESRD when GFR<30ml/min/1.73m^2^ (bottom panel), while the ESRD risk associated to GFR decline (negative ΔGFR) was attenuated from stage 4–5 to stage 3a or higher (see the Fig 3 from bottom to the top).

## Discussion

This multicenter cohort study in stage 3–4 CKD patients on long term follow up in Nephrology clinics originally shows that CKD regression, occurs in about one-quarter of cases, it is independent of CKD stage andpredicts a lower risk of ESRD, while not being associated with excess mortality.

The lower incidence of ESRD among CKD regressors (1% per year) is only apparently obvious. Indeed, the change of renal function over time varies considerably and shows a non-linear decline in up to 40% of CKD patients [29]; therefore, upward eGFR fluctuations over relatively short periods do not necessarily predict better renal outcomes. By in-depth analysis of this association, we evidenced that renal prognosis in regressors was influenced by the stage of CKD at the start of survival analysis. When considering the level of eGFR reached by patients at the end of the period used for definition of ΔGFR, regressor status was overall associated with a lower risk of ESRD independently from the CKD stage reached (Table 4); however, patients that at the end of eGFR improvement persisted in stage 4 remained exposed to a relatively greater ESRD risk (Fig 3).

Variability of eGFR in our CKD population is likely attenuated because referred patients under stable nephrology care represent a selected population, that is, more homogeneous as compared to the general CKD population. The cut-off level chosen for defining CKD regressors (ΔGFR≥0 ml/min/1.73 m² within at least two years) was a pragmatic, predefined cut-off which was not based on strict, rigourous methodology like the one applied in the AASK trial population [4]. However a loss of 1 ml/min/year is universally considered as kidney loss in healthy subjects [16–18]; therefore it is possible to consider as regression also a ΔGFR equal to 0. Yet this pragmatic cut-off is seemingly adequate to predict an excellent renal prognosis, suggesting that the condition of CKD regressor heralds a true, long term, improvement in renal function also outside the trial setting. A recent meta-analysis [15], aimed at assessing the association of ΔGFR with subsequent risk of ESRD, has similarly reported an association between improved eGFR and lower risk of ESRD. At variance with that study, however, we also considered proteinuria, causes of CKD, BMI, phosphoremia, uricemia and Hb, as potential confounders of CKD regression. Nephrologist care of CKD patients is associated with better renal outcomes as compared to primary care or other settings [30,31]. Nevertheless, in studies focusing on the relevance of specialist renal care for the prevention of kidney failure very scanty attention has been dedicated to CKD regression. Improvement in renal function in CKD patients was only incidentally reported in observational studies carried out in settings different from nephrology clinic, such as general population [8,9,32], primary care [7] and patients with rheumatoid arthritis [6]. In these studies, the entity of eGFR improvement varied widely, from 3% to 33% [4,6–9,32], which likely depends on differences in study design (retrospective or prospective), baseline renal function, inclusion/exclusion criteria, ethnicity and demographic characteristics.

The definition of chronicity of CKD is critical in studies looking at the prognostic value of GFR changes over time. Accordingly, we defined chronicity by a stringent, conservative criterion, i.e., when the diagnosis of CKD was well documented at least 1 year before of study entry. This is important because prolonging the chronicity criterion for CKD definition from 3 to 12 months substantially reduces the number of patients with true CKD [33]. Furthermore, since our cohort included patients under stable nephrology care without any recent acute change in eGFR, it is unlikely that short-term functional or hemodynamic worsening of renal function, may have accounted for the subsequent eGFR improvement.

Due to the regression-to-the mean phenomenon, the method we used to assess eGFR change over time, that is, a two-point evaluation, is inherently prone to overestimate the prevalence of CKD regressors. However, this estimate was confirmed in a sensitivity analysis performed in the 82% of the whole cohort where ≥3 eGFR measurements were available during follow up. Al-Aly et al, reported that 38% of 4171 patients with early CKD (eGFR 59–45 ml/min) maintained stable kidney function over a 2.6 year follow-up and similar results were registered by Eriksen et al in CKD stage 3 [6, 32]. These observations suggest that eGFR stability or improvement may be a peculiarity of earlier CKD stages. In line with these findings we also found that the prevalence of CKD regressors in stage 3a was higher than at more advanced CKD stages, even though after multiple adjustments, this association disappeared after multiple adjustments.

Our analysis identified low proteinuria as the strongest factor associated with regressor status. This factor, measured by gold standard, accounted for almost half of the variance of the corresponding logistic model, therefore extending to Caucasian patients the findings obtained in the AASK population [4]. However, in our study the association beween proteinuria and the probability of CKD regressor status was not linear and indicated exponentially decreasing odds of regression from 0.5 g/day on (Fig 2) with the only exception of PKD that was associated with worse renal prognosis independent of proteinuria; this finding may be related to the lower responsiveness to any nephroprotective treatment of these patients [34,35]. We observed that proteinuria increased in regressors and decreased in non regressors. This difference was of minor entity and, at least in part, dependent on the similar changes in GFR, that increased in regressors and decreased in non regressors.

The lack of relationship between CKD regression and mortality in the present study apparently contrasts with studies reporting a paradoxical link between the eGFR rise and the greater mortality risk [6–9]. Our results may depend on the fact that mortality in CKD patients cared by nephrologists is lower than in CKD patients not receiving specialist renal care, as previously reported [10,36,37]. Furthermore, in previous studies the predominance of male sex [6,9], older age [7], black race [6–8] and coronary heart disease [6,8] in apparent CKD regressors may have accounted for higher mortality risk. Finally, the improvement in eGFR in these studies may be at least in part dependent on a decrease in serum creatinine as a result of muscle loss related to coexisting comorbid illness or malnutrition [6–8], a phenomenon which seems unlikely in our cohort, as no reduced BMI or serum albumin was registered in CKD regressors.

The present study has limitations. The observational design precludes interpretation of results in causal terms. Furthermore, our findings cannot be generalized to unreferred patients or to ethnic groups other than Caucasians. Moreover, we cannot exclude survival bias that may be present in referred cohorts, and partly suggested by older age and higher prevalence of CVD of patients died during the window time (18–24 months) for evaluation of regressor status. Finally, serum creatinine was not calibrated thus possibly reducing the adequacy of GFR estimates [38,39]; however, we adopted an established calibration factor to correct creatinine measurement not standardized to isotope dilution mass spectrometry in order to perform CKD-EPI creatinine equation [15,21].

In conclusion, CKD regression occurs in one-fourth of CKD patients receiving outpatient nephrology care especially in the absence of high proteinuria, hypertension and diagnosis of PKD. Identification of CKD regressors has prognostic relevance because these patients exhibit lower risk of ESRD and no higher risk of death. This phenomenon may be therefore considered in conjunction with main risk factors, including eGFR level reached by patients, in order to optimize renal risk stratification.

## Supporting Information

S1 Dataset Excel format. (XLSX)

## References

[1] Kidney Disease: Improving Global Outcomes (KDIGO) CKD Work Group. KDIGO 2012 Clinical Practice Guideline for the Evaluation and Management of Chronic Kidney Disease. Kidney Int Suppl. 2012;3:1–150.

[2] Remuzzi G, Benigni A, Remuzzi A. Mechanisms of progression and regression of renal lesions of chronic nephropaties and diabetes. J Clin Invest 2006, 116(2): 288–96 16453013 10.1172/JCI27699 PMC1359063

[3] Ruggenenti P, Perna A, Benini R, Bertani T, Zoccali C, Maggiore Q, et al In chronic nephropathies prolonged ACE inhibition can induce remission: dynamics of time-dependent changes in GFR. Investigators of the GISEN Group. Gruppo Italiano Studi Epidemiologici in Nefrologia, J Am Soc Nephrol 1999, 10(5):997–1006. 10232685 10.1681/ASN.V105997

[4] Hu B, Gadegbeku C, Lipkowitz MS, Rostand S, Lewis J, Wright JT, et al for the African-American Study of Kidney Disease and Hypertension Group. Kidney function can improve in patients with hypertensive CKD. J Am Soc Nephrol 2012;23: 706–13. 22402803 10.1681/ASN.2011050456 PMC3312500

[5] Weis L, Metzger M, Haymann JP, Thervet E, Flamant M, Vrtovsnik F, et al Renal function can improve at any stage of chronic kidney disease. PLoS One 2013; 8(12): e–81835 10.1371/journal.pone.0081835 PMC3862566 24349134

[6] Al-Aly Z, Zeringue A, Fu J, Rauchman MI, McDonald JR, El-Achkar TM, et al Rate of kidney function decline associates with mortality. J Am SocNephrol 2010;21:1961–9. 10.1681/ASN.2009121210 PMC3014010 20947634

[7] Perkins RM, Bucaloiu ID, Kirchner HL, Ashouian N, Hartle JE, Yahya T. GFR decline and mortality risk among patients with chronic kidney disease. Clin J Am SocNephrol 2011;6:1879–86. 10.2215/CJN.00470111 PMC3359538 21685022

[8] Turin TC, Coresh J, Tonelli M, Stevens PE, de Jong PE, Farmer CK, et al Change in the estimated glomerular filtration rate over time and risk of all-cause mortality. Kidney Int 2013; 83:684–91. 10.1038/ki.2012.443 23344477

[9] Matsushita K, Selvin E, Basch LD, Franceschini N, Astor BC, Coresh J. Change in estimated GFR associated with coronary heart disease and mortality. J Am Soc Nephrol 2009; 20(12): 2617–24. 10.1681/ASN.2009010025 19892932 PMC2794225

[10] De Nicola L, Chiodini P, Zoccali C, Borrelli S, Cianciaruso B, Di Iorio B, et al SIN-TABLE CKD Study Group. Prognosis of CKD patients receiving outpatient nephrology care in Italy. Clin J Am SocNephrol 2011; 6(10): 2421–8. 10.2215/CJN.01180211 PMC3359552 21817127

[11] Leonardis D, Mallamaci F, Enia G Postorino M, Tripepi G, Zoccali C, et al The MAURO study: baseline characteristics and compliance with guidelines targets. J Nephrol 2012; 25(06): 1081–90. 23172127 10.5301/jn.5000239

[12] Kidney Disease Outcomes Quality Initiative (K/DOQI). K/DOQI clinical practice guidelines on hypertension and hypertensive agents in chronic kidney disease. Am J Kidney Dis 2004;43:S1–S290. 15114537

[13] Cianciaruso B. Conservative therapy Guidelines for chronic renal failure. G Ital Nefrol 2003; 20 (Suppl 24): S48–S60. 14666503

[14] Mallamaci F, Minutolo R, Leonardis D, D'Arrigo G, Tripepi G, Rapisarda F, et al Long-term visit to visit office blood pressure variability increases the risk of adverse cardiovascular outcomes in patients with chronic kidney disease. Kidney Int 2013; 84: 381–9. 10.1038/ki.2013.132 23615498

[15] Coresh J, Turin TC, Matsushita K, Sang Y, Ballew SH, Appel LJ, et al for the CKD Prognosis Consortium. Decline in Estimated Glomerular Filtration Rate and Subsequent Risk of End-Stage Renal Disease and Mortality. JAMA. 2014; 311:2518–31. 24892770 10.1001/jama.2014.6634 PMC4172342

[16] Lindeman RD, Tobin J, Shock NW. Longitudinal studies on the rate of decline in renal function with age. J Am Geriatr Soc 1985; 33:278–85. 3989190 10.1111/j.1532-5415.1985.tb07117.x

[17] Poggio ED, Rule AD, Tanchanco R, Arrigain S, Butler RS, Srinivas T, et al Demographic and clinical characteristics associated with glomerular filtration rates in living kidney donors. Kidney Int 2009; 75:1079–87 10.1038/ki.2009.11 19212414 PMC2713659

[18] Wetzels JF, Kiemeney LA, Swinkels DW, Lems HL, den Heijer M. Age- and gender-specific reference values of estimated GFR in Caucasians: the Nijmegen Biomedical Study. Kidney Int 2007; 72:632–7. 17568781 10.1038/sj.ki.5002374

[19] Mancia G, De BG, Dominiczak A, Cifkova R, Fagard R, Germano G, et al Guidelines for the management of arterial hypertension: the task force for the management of arterial hypertension of the European Society of Hypertension (ESH) and of the European Society of Hypertension (ESC), J Hypertension 2007; 25: 1105–87. 10.1097/HJH.0b013e3281fc975a 17563527

[20] Levey AS, Coresh J, Green T, Stevens LA, Zhang YL, Hendriksen S, et al Using standardized serum creatinine values in the modification of diet in renal disease study for estimating glomerular filtration rate. Ann Int Med 2006; 145:247–54. 16908915 10.7326/0003-4819-145-4-200608150-00004

[21] Levey AS, Stevens LA, Schmid CH, Zhang YL, Castro AF 3rd, Feldman HI, et al A new equation to estimate glomerular filtration rate. Ann Intern Med. 2009;150(9):604–12. 19414839 10.7326/0003-4819-150-9-200905050-00006 PMC2763564

[22] Levey AS, Coresh J, Greene T, Marsh J, Stevens LA, Kusek JW, et al Expressing the Modification of Diet in Renal Disease Study equation for estimating glomerular filtration rate with standardized serum creatinine values. Clin Chem. 2007;53(4):766–72. 17332152 10.1373/clinchem.2006.077180

[23] Dwyer J, Kenler SR. Assessment of nutritional status in renal disease In: Nutrition and the Kidney, edited by Mitch WE, Klahr S, 2nd Ed., Boston: Little, Brown and Company, 1993, pp 61–95

[24] Tjur T. Coefficients of determination in logistic regression models—A new proposal: the coefficient of discrimination. The American Statistician 2009; 63:366–72.

[25] Harrell FE Jr, Lee KL, Pollock BG. Regression models in clinical studies: determining relationships between predictors and response. J Nat Cancer Inst 1988; 80:1198–2202. 3047407 10.1093/jnci/80.15.1198

[26] Schemper M, Smith TL. A note on quantifying follow-up in studies of failure time. Control Clin Trials 1996; 17:343–6. 8889347 10.1016/0197-2456(96)00075-x

[27] Glymour MM, Weuve J, Berkman LF, Kawachi I, Robins JM.When is baseline adjustement useful in analyses of change? An example with education and cognitive change. Am J Epidemiol 2005; 162: 267–78. 15987729 10.1093/aje/kwi187

[28] Landis JR, Koch GG. The measurement of observer agreement for categorical data. Biometrics 1977; 33:159–74. 843571

[29] Li L, Astor BC, Lewis J, Hu B, Appel LJ, Lipkowitz MS, et al Longitudinal progression trajectory of GFR among patients with CKD. Am J Kidney Dis. 2012; 59:504–512. 10.1053/j.ajkd.2011.12.009 22284441 PMC3312980

[30] Jones C, Roderick P, Harris S, Rogerson M. Decline in kidney function before and after nephrology referral and the effect on survival in moderate to advanced chronic kidney disease. Nephrol Dial Transplant 2006; 21(8): 2133–43. 16644779 10.1093/ndt/gfl198

[31] Tseng CL, Kerne EF, Miller Dr, Tiwari A, Maney M, Rajan M, et al Survival benefit of nephrologic care in patients with diabetes mellitus and chronic kidney disease. Arch Intern Med 2008; 168: 55–62. 10.1001/archinternmed.2007.9 18195196

[32] Eriksen BO, Ingebretsen OC. The progression of chronic kidney disease: A 10-year population-based study of the effects of gender and age. Kidney Int 2006; 69:375–82. 16408129 10.1038/sj.ki.5000058

[33] Eriksen BO, Ingebretsen OC. In chronic kidney disease staging the use of the chronicity criterion affects prognosis and the rate of pregression. Kidney Int 2007; 72:1242–8. 17687256 10.1038/sj.ki.5002472

[34] Ecder T, Chapman AB, Brosnahan GM, Edelstein CL, Johnson AM, Schrier RW. Effect of antihypertensive therapy on renal function and urinary albumin excretion in hypertensive patients with autosomal dominant polycystic kidney disease. Am J Kidney Dis 2000;35:427–32. 10692268 10.1016/s0272-6386(00)70195-8

[35] Hogan MC, Masyuk TV, Page LJ, Kubly VJ, Bergstralh EJ, Li X, et al Randomized clinical trial of long-acting somatostatin for autosomal dominant polycystic kidney and liver disease. J Am Soc Nephrol 2010; 21:1052–61. 10.1681/ASN.2009121291 20431041 PMC2900957

[36] De Nicola L, Minutolo R, Chiodini P, Borrelli S, Zoccali C, Postorino M, et al The effect of increasing age on the prognosis of non-dialysis patients with chronic renal disease receiving stable nephrology care. Kidney Int 2012; 82 (4): 482–8. 22622495 10.1038/ki.2012.174

[37] Minutolo R, Lapi F, Chiodini P, Borrelli S, Zoccali C, Postorino M, et al Risk of ESRD and death in patients with CKD not referred to a nephrologist: a 7-year prospective study. Clin J Am Soc Nephrol. 2014 9 5;9(9):1586–93. 10.2215/CJN.10481013 25074838 PMC4152817

[38] Stevens LA, Manzi J, Levey AS, Chen J, Deysher AE, Greene T, et al Impact of creatinine calibration on performance of GFR estimating equations in a pooled individual patient database. Am J Kidney Dis 2007;50:21–35 17591522 10.1053/j.ajkd.2007.04.004

[39] Murthy K, Stevens LA, Stark PC, Levey AS. Variation in the serum creatinine assay calibration: A practical application to glomerular filtration rate estimation. Kidney Int 2005; 68:1884–7. 16164667 10.1111/j.1523-1755.2005.00608.x

